# Biological therapies and management of oral mucosal disease

**DOI:** 10.1038/s41415-024-7065-9

**Published:** 2024-02-23

**Authors:** Claire M. Healy, Sheila Galvin

**Affiliations:** 4141518816001 Consultant/Professor in Oral Medicine, Dublin Dental University Hospital, School of Dental Science, Trinity College Dublin, Ireland; 4141518816002 Consultant/Assistant Professor in Oral Medicine, Dublin Dental University Hospital, School of Dental Science, Trinity College Dublin, Ireland

## Abstract

Biologic drugs are drugs made by living organisms and the term is usually limited to monoclonal antibodies or receptors targeting specific cytokines or cells that have been developed in recent decades. These drugs have had an enormous impact on the management of cancers, including head and neck cancers, and immune-mediated inflammatory conditions, for example, rheumatoid arthritis and inflammatory bowel disease. General dental practitioners will routinely be managing patients who are on these medications for a wide range of systemic conditions. These drugs also have a limited role in the management of immune-mediated oral mucosal disease. In this article, we will introduce the range of biological agents and their systemic indications and then elaborate on their use in oral mucosal disease and the disadvantages associated with their use.

## Key points

- General dental practitioners will increasingly manage patients on biologic therapy for a wide range of systemic indications. This paper is a useful introduction to these biological therapies.
- Biologic therapy has a limited role in the management of recalcitrant immune-mediated oral mucosal disease, in which the use of most of these agents is off-label.
- The most convincing evidence for their use in oral disease relates to rituximab, a B-cell inhibitor, which is licenced for use in pemphigus vulgaris and is now a first-line agent in the management of this disease.

## Introduction

Biological therapy is the management of disease with a substance that is produced by a living organism. It has revolutionised the management of various cancers, such as the use of the biologic checkpoint inhibitors, pembrolizumab and nivolumab in the management of advanced head and neck cancers. In addition, it has had an enormous impact on the management of immune-mediated diseases through its ability to directly target specific pathways, cytokines or proteins involved in the disease process. There has been a recent explosion in the development of biologic agents for immune-mediated disease due to an increased understanding of disease pathogenesis, which has resulted in an expansion in the range of targets and indications for these medications. Furthermore, biological therapies are also increasingly being used in the management of immune-mediated diseases of the oral mucosa.

## Classes of biological therapies

The main classes of biological therapies in widespread use include: i) anti-tumour necrosis factor-alpha (TNF-α) inhibitors; ii) anti-interleukin (IL) therapies; iii) anti-integrin inhibitors; iv) anti-B cell inhibitors; and v) anti-T cell inhibitors (Table 1). Many of these agents are monoclonal antibodies, denoted by the suffix -mab, that target a specific cytokine, for example, infliximab which targets TNF- α, or a cell marker, for example, rituximab which targets CD20 which is expressed solely on B cells. Others act as soluble receptors, for example, etanercept, which binds TNF-α and prevents its pro-inflammatory actions. These drugs are delivered either via intravenous infusion or subcutaneous injection.

**Table 1:** Commonly used biological therapies with their targets and indications, several of which have oral manifestations

Drug	Common trade names	Target	Indication
Adalimumab	Humira, Hulio	TNF-α	Crohn's
Infliximab	Remicade, Flixabi	TNF-α	Crohn's
Golimumab	Simponi	TNF-α	RA, PA, ankylosing spondylitis
Certolizumab	Cimzia	TNF-α	Crohn's, RA, PA, ankylosing spondylitis
Etanercept	Enbrel, Benepali	TNF-α	Plaque psoriasis, RA, PA, ankylosing spondylitis, JIA
Ustekinumab	Stelara	IL-12, IL-23	Crohn's, UC, plaque psoriasis, PA
Vedolizumab	Entyvio	α4β7 integrin	Crohn's, UC
Rituximab	Rituxan	CD20	Multiple - RA, NHL, CLL, GPA, pemphigus
Abatacept	Orencia	CD80, CD86	RA, JIA, PA
Key: RA = Rheumatoid arthritis; PA = Psoriatic arthritis; JIA = Juvenile idiopathic arthritis; UC = ulcerative colitis; NHL = Non-Hodgkin's lymphoma; CLL = Chronic lymphocytic leukaemia; GPA = Granulomatosis with polyangiitis			

TNF-α is perhaps the most significant regulator of inflammation and is involved in the pathogenesis of many inflammatory and autoimmune conditions. Anti-TNF therapies were among the first biologics to be developed and include infliximab, adalimumab, golimumab, certolizumab pegol and etanercept.^1^ Infliximab, adalimumab and golimumab are anti-TNF-α monoclonal antibodies, certolizumab pegol is a monoclonal antibody fragment specific to TNF-α, while etanercept is a TNF-α receptor fusion protein. These drugs are widely used in the management of rheumatoid and psoriatic arthritis (RA/PA), ankylosing spondylitis and Crohn's disease.

Ustekinumab is a biological therapy that targets the interleukins, IL-12 and IL-23. It acts by inhibiting their binding to their receptors on T lymphocytes and natural killer cells thus preventing IL-12 and IL-23-mediated T-cell activation and cytokine production.^2^ Ustekinumab is used in the management of psoriasis, PA, ulcerative colitis (UC) and Crohn's disease.

Vedolizumab is an anti-α4β7 integrin used in the management of inflammatory bowel disease and may reduce gastro-intestinal inflammation by inhibiting T-cell recruitment to the intestine, although its precise mechanism of action is unclear.^3^

Rituximab is a chimeric murine/human monoclonal antibody targeting the CD20 antigen found on the surface of B-cells and so acts to deplete the circulating B-cell population.^4^ It was initially developed for use in B cell malignancies, for example, non-Hodgkin's lymphoma and chronic lymphocytic leukaemias, but its use rapidly extended to B-cell mediated autoimmune disorders, for example, granulomatosis with polyangiitis and RA.

Finally, abatacept is a biologic T-cell inhibitor which binds to CD80 and CD86 molecules on antigen-presenting cells, thus preventing their interaction with T-cell CD28 and therefore T-cell activation.^5^ It is used in the management of T-cell-mediated conditions such as RA and PA.

## Use in oral mucosal disease

### Orofacial granulomatosis and oral Crohn's disease

Biologic therapy, specifically anti-TNF therapy, has been the mainstay of management of Crohn's disease for many years. Off-label anti-TNF therapy has also been used successfully in the management of recalcitrant orofacial granulomatosis (OFG). Elliott *et al.* reported the outcomes of treatment in a series of 14 patients with orofacial granulomatous disease, seven of whom had OFG, with the remaining seven having oral and intestinal Crohn's disease.^6^ In total, 71% had a short-term response to infliximab, while 33% remained responsive at two years. Two of those who failed to respond to infliximab responded to adalimumab. A recent European multicentre case series of 28 patients with oral Crohn's disease included ten who were commenced on anti-TNF therapy.^7^ Nine of these patients achieved disease remission with anti-TNF therapy (infliximab or adalimumab), and in another, adalimumab achieved disease remission but had to be stopped due to side effects. This latter patient went on to have a sustained response with vedolizumab. Another patient, whose initial response to infliximab was not maintained and who failed to respond to vedolizumab, went on to respond to ustekinumab. Success with ustekinumab in the management of oral Crohn's has been reported in isolated cases by others also.^8^^,^^9^

An important complication of the use of biological agents is the risk of infection and a case of perioral cellulitis associated with the use of adalimumab in OFG has been reported. The diagnosis of cellulitis can be delayed as the clinical manifestations are similar to those of OFG and this diagnosis should be borne in mind if lip/facial swelling is spreading, becomes hot or tender, or is associated with systemic symptoms.^10^

### Pemphigus

**Fig. 1:**
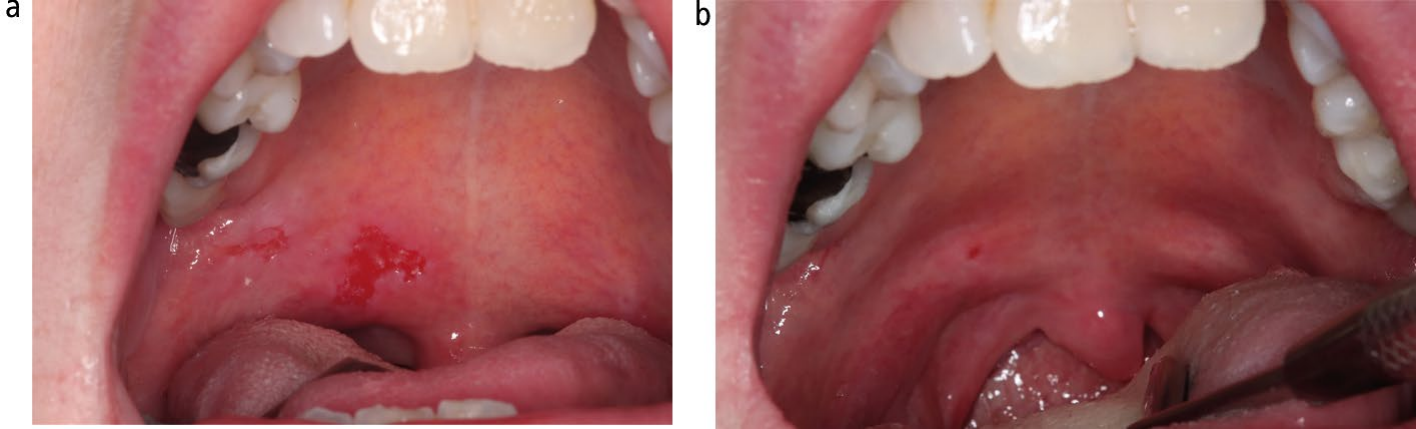
a, b) Clinical photographs showing the soft palate of a 48-year-old female patient with both oral and skin pemphigus vulgaris pre- and post-rituximab therapy. Extensive palatal erosion uncontrolled by conventional immunosuppression is evident (left) which came substantially under control following rituximab infusion (right)

Pemphigus vulgaris (PV) is a B-cell mediated autoimmune disorder, whose pathogenicity is due to the production of anti-desmoglein antibodies targeting the desmosomal proteins - desmoglein 1 and desmoglein 3 - found predominantly on skin and mucosa, respectively. The treatment of pemphigus has traditionally involved corticosteroids which are very effective in gaining rapid control of the disease, but protracted courses are associated with many, well-established side effects. Steroid-sparing agents such as mycophenolate mofetil and azathioprine have played an important role in allowing the reduction or cessation of corticosteroids in these patients. However, the advent of rituximab has revolutionised the management of the disease, allowing a precision approach targeting the production of the pathogenic antibodies (Fig. 1).

The Ritux-3 study demonstrated the effectiveness of rituximab as a first line agent in the management of pemphigus.^11^ In total, 89% of 46 patients treated with 3-6 months of tapering doses of systemic corticosteroids (0.5-1.0 mg/kg) and rituximab (1 g delivered at 1 and 14 days, 500 mg delivered at 12 and 18 months) were in complete remission at 24 months, while only 34% of 44 patients treated with higher tapering doses of corticosteroids (1.0-1.5 mg/kg) for 12-18 months were in complete remission at this point. Furthermore, significantly more patients in the corticosteroid-alone group had complications with diabetes and myopathy because of the necessity for higher steroid doses in the absence of rituximab. Both groups had similar numbers of treatment-related infections; however, three patients from the rituximab group developed significant infections, while one patient in the high-dose corticosteroid group did. All infections were treatable and did not necessitate the cessation of immunosuppression.

Rituximab is licenced for use in pemphigus in the UK by the Medicines and Healthcare products Regulatory Agency, in the European Union by the European Medicines Agency and in the USA by the Food and Drugs Administration agency. It is recommended as a first line agent in the management of pemphigus along with corticosteroids by the European Academy of Dermatology and Venereology and by an international panel of pemphigus experts.^12^^,^^13^

While the role of rituximab has been clearly established in the management of pemphigus, other biological agents in common usage have not been shown to be of significant benefit. A small randomised controlled trial (RCT) (20 patients in total) investigated the effect of infliximab in steroid-dependent PV and, while infliximab was associated with a reduction in titres of anti-desmoglein antibodies, this did not correlate into a significant clinical effect, which may relate to the low power of the study.^14^ A novel biologic agent, efgartigimod, has recently been investigated in PV and pemphigus foliaceous (PF) in a phase II clinical trial (NCT03334058). This agent binds endogenous FcRn (neonatal Fc receptor for IgG) which functions to recycle IgG and so efgartigimod results in the degradation of IgG. It has shown early promise in myasthenia gravis, another antibody-mediated disease. Initial results in PV and PF have been positive and the clinical response has been reflected in a reduction in anti-desmoglein antibody levels.^15^^.^^16^

### Mucous membrane pemphigoid

While there are numerous case series, there are no RCTs reporting the use of biologic agents in mucous membrane pemphigoid (MMP). However, results are awaited of a French multicentre RCT with an estimated completion date of November 2023 comparing the safety and efficacy of rituximab and oral cyclophosphamide in the management of severe MMP (https://clinicaltrials.gov/ct2/show/NCT03295383).

A recently published retrospective review from a single centre in France reported the unit's experience with rituximab in 109 patients with severe MMP (61.5% of whom had oral involvement) over a ten-year period.^17^ Rituximab was used in patients with severe refractory disease who failed to respond to conventional immunosuppression (for example, mycophenolate mofetil, cyclophosphamide) or in whom such immunosuppression was contra-indicated and generally involved two 1 g infusions 14 days apart, repeated at six-monthly intervals until complete remission or treatment failure. At the start of rituximab therapy, most patients remained on some immunomodulatory therapy (for example, dapsone, tetracyclines), while fewer remained on a stable dose of topical or systemic corticosteroids. Complete remission was achieved in 85.3% of patients after two cycles of rituximab. One year after discontinuation of rituximab, complete remission was maintained in 68.7%.

A recent systematic review looking at the outcomes of biologic treatments in patients with MMP included 63 studies with 331 patients, 39% of whom had oral involvement.^18^ The studies were heterogeneous in nature and only 20.8% were on biologic monotherapy making interpretation of findings difficult. The therapies included intravenous immunoglobulin (IVIG), a commonly used treatment modality in autoimmune disorders, comprising pooled concentrate of immunoglobulins from healthy donors. Of 154 patients treated with IVIG, 61.7% had complete remission, while of those treated with rituximab (n = 112, 70.5%) had complete remission. Interestingly, while IVIG worked more slowly, it was associated with a lower risk of disease recurrence than rituximab. Only seven patients were treated with anti-TNF therapy and 71% of these developed complete remission and did so more quickly than with either other agent. Of note, rituximab was associated with the most significant adverse reactions, with two patients dying from severe infection.

Recent European guidelines on the diagnosis and management of MMP have recommended rituximab as a second line agent in severe MMP and as a third line agent in mild/moderate MMP that is unresponsive to conventional immunosuppression.^19^ IVIG was recommended as a third line agent in severe MMP, while anti-TNF therapy was recommended as a fourth line agent, the latter on the foot of the low number of reports on anti-TNF therapy in the MMP literature as evidenced in Lytvyn *et al.*'s systematic review.^18^

### Lichen planus

While there are isolated case reports of the use of biologic agents in the management of lichen planus (LP) reporting limited success, there are no substantial case series or RCTs in the literature to support their use. LP is a T-cell-mediated disease and one small case series (seven patients) of a biologic that selectively targets T memory cells (alefacept) suggested it may result in symptomatic improvement in some LP patients,^20^ but the drug was subsequently withdrawn from the market. Interestingly, there are numerous reports in the literature of lichenoid reactions to biologics prescribed for other purposes, including infliximab,^21^^,^^22^ rituximab^23^^,^^24^ and dupilumab,^25^ which may have limited the use of biologics in LP. An added factor is the risk of malignant transformation in LP, which could potentially be enhanced by the use of biologic agents.

### Recurrent aphthous stomatitis

Recurrent aphthous stomatitis (RAS) is the most common oral mucosal disease and generally responds well to addressing predisposing factors and the use of topical corticosteroids and antibacterial and analgesic mouth rinses. However, some patients may have recalcitrant RAS requiring systemic immunosuppression. The role of biologic agents in recalcitrant RAS was the subject of a recent review,^26^ which highlighted the very few isolated case reports in the literature of these agents in RAS in the absence of a systemic disease, and there is insufficient evidence to support their use.

However, there is more, though limited, evidence for their use in RAS associated with systemic disease. Behcet's disease is a chronic multi-system vasculitis characterised by recurrent oral ulceration which can have a significant impact on patient quality of life. While most patients will respond to topical corticosteroids and colchicine, there are some who fail to respond to these simple measures. Recommendations from the European League against Rheumatism have supported the use of TNF-α inhibitors in such patients.^27^ The IL-1 inhibitors, anakinra (IL-1 receptor antagonist) and canakinumab (anti-IL-1β monoclonal antibody), and the IL-12 and IL-23 inhibitor, ustekinumab, may also have roles to play, although the studies investigating these agents involved very small numbers of patients.^28^^,^^29^

RAS is also a common manifestation of Crohn's disease, an inflammatory bowel disease in which biologic agents are frequently used. A meta-analysis was recently carried out looking at the prevalence of RAS in patients with inflammatory bowel disease who were being treated with anti-TNF therapy or anti-integrin therapy.^30^ It included six studies of patients with Crohn's disease (n = 1,477) and four of patients with UC (n = 267). Anti-TNF therapy resulted in a greater reduction in aphthous ulceration in both Crohn's disease and UC than anti-integrin therapy, but it was acknowledged that this may reflect a more severe disease phenotype in the anti-integrin cohort, as these are generally second line agents.

### Circumorificial plasmacytosis

**Fig. 2:**
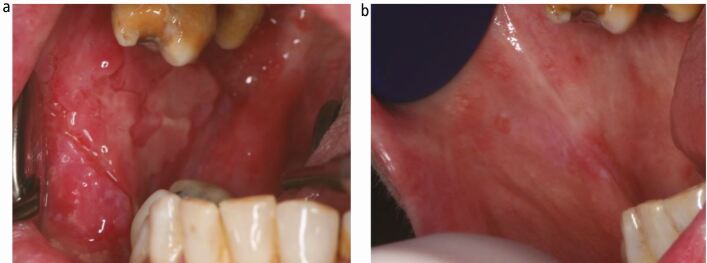
a, b) Clinical photographs showing the right buccal mucosa in a 66-year-old female patient with circumorificial plasmacytosis pre- and post-adalimumab therapy. She had extensive debilitating oral involvement which was unresponsive to conventional immunosuppression, but rapidly came under control with the introduction of adalimumab fortnightly. She continues to need maintenance adalimumab

Circumorificial plasmacytosis, also known as plasma cell orificial mucositis, is a rare chronic plasma cell proliferative disorder of the orificial mucous membranes of unknown aetiology. There are few cases reported in the literature and no established treatment to date. However, a small case series from our unit included a case which resolved with treatment with adalimumab (Fig. 2).^31^

## Disadvantages with biologic use

Biologics are commonly associated with headaches, malaise, nausea and arthralgia. Unsurprisingly, their most significant side effect is infection, for example, urinary tract infections, pneumonia and deaths can occur due to sepsis and COVID-19 infection. Progressive multifocal leukoencephalopathy due to JC virus infection is also a significant, albeit very rare, side effect of rituximab therapy. Prior to commencing biologic therapy, patients need to be screened for occult infection, including human immunodeficiency virus, hepatitis B and C, and tuberculosis, and for immunity to varicella zoster, and need vaccination against influenza, SARS-CoV-2 and pneumococcus.

As the immune system plays a key role in cancer surveillance, when biologics were initially introduced, there were concerns about a possible increased risk of malignancy, including oral cancer.^32^ However, reassuringly, most recent large-scale studies indicate there is not a significantly increased risk of malignancy, apart from non-melanoma skin cancer.^33^^,^^34^^,^^35^ Nonetheless, vigilance is advised and patients should engage with national cancer screening programmes, for example, cervical, breast and bowel, and need to avoid sun exposure and use sun protection.

From an oral standpoint, in addition to being associated with some oral infections, for example, herpes simplex and candida, biologics, as mentioned, can cause oral lichenoid reactions.^21^^,^^22^^,^^23^^,^^24^^,^^25^ Paradoxically, they have also been reported to be a rare trigger for immunobullous disease.^36^

Biologic agents are expensive, for example, the initial infusions of rituximab given two weeks apart cost approximately £3,000, with the additional infusions at 12 and 18 months costing approximately £500 each. Furthermore, many biologics, including rituximab, need to be administered by intravenous infusion in a hospital setting with resuscitation facilities, while others can be self-delivered by the patient by subcutaneous injection following appropriate training. Their use in oral mucosal disease necessitates liaison with other specialties, for example, dermatology and immunology, as they are not suitable for administration in a dental hospital setting.

## Future directions

Chimeric antigen receptor T-cell (CAR-T) therapy has recently been used with great success in the management of haematological malignancies and now, chimeric auto-antibody receptor T-cell (CAAR-T) therapy is being explored in some autoimmune diseases. This is a form of biologic therapy where a patient's own T-cells are engineered to express receptors that will target cells bearing particular antigens, which, when re-infused, will result in direct T-cell-mediated killing of these cells. The DesCAARTes phase 1 clinical trial is investigating CAAR-T therapy in pemphigus, in which patient T-cells are being engineered to recognise anti-desmoglein 3, which is expressed by B-cells that produce anti-desmoglein 3 (https://clinicaltrials.gov/ct2/show/NCT04422912). This allows a very precise approach as only pathogenic B-cells will be targeted and so it is expected that there will be reduced side effects, in particular, infections. The trial is currently recruiting patients with active mucosal PV in several centres in the USA and the study is expected to be completed in September 2026.

## Conclusion

While biological therapies are being used increasingly in systemic immune-mediated disease, they still have a limited role in the management of oral mucosal diseases. Most immune-mediated oral diseases can be managed effectively by optimal use of topical and conventional systemic immunosuppression, with antiseptic and analgesic mouth rinses and good oral hygiene being important adjuncts. However, biological therapy does play an important role in the management of some oral diseases, in particular, rituximab in pemphigus and anti-TNF-α agents in recalcitrant OFG. Apart from the use of rituximab in pemphigus and various agents in Crohn's disease, the use of biologics in oral mucosal disease is off-label. Patients requiring their use need education and screening before their introduction and careful monitoring. While biologic agents do not impact directly on the delivery of dental care, dentists should be aware of the increased susceptibility to severe infection of patients on these therapies.
