# Correction to: Biological therapies and management of oral mucosal disease

**DOI:** 10.1038/s41415-024-7342-7

**Published:** 2024-04-26

Journal's correction note:

General article *Br Dent J* 2024; **236:** 317-321.

When initially published online, the corresponding author metadata was erroneously inputted, leading to the name being incorrectly indexed as ‘M Healy C' instead of ‘Healy C M'. We have now corrected this to ensure the corresponding author name is indexed correctly.

The journal apologises for any inconvenience caused.

